# The Intra-Articular Delivery of a Low-Dose Adeno-Associated Virus-IL-1 Receptor Antagonist Vector Alleviates the Progress of Arthritis in an Osteoarthritis Rat Model

**DOI:** 10.3390/pharmaceutics16121518

**Published:** 2024-11-25

**Authors:** Shuang Luo, Hao Jiang, Qingwei Li, Shiping Yang, Xuemei Yu, Xiongliang Xu, Qing Xie, Xiao Ke, Qiang Zheng

**Affiliations:** 1 Chengdu Origen Biotechnology Co., Ltd., Chengdu 610036, China; luoshuang@cnkh.com (S.L.); jianghao@cnkh.com (H.J.); liqingwei@cnkh.com (Q.L.); 022228@cnkh.com (S.Y.); 015816@cnkh.com (X.Y.); 013191@cnkh.com (X.X.); 023562@cnkh.com (Q.X.); 2 Therapeutic Proteins Key Laboratory of Sichuan Province, Chengdu 610037, China; 3 Chengdu Kanghong Pharmaceuticals Group Co., Ltd., Chengdu 610037, China

**Keywords:** osteoarthritis, gene therapy, adeno-associated virus, IL-1Ra

## Abstract

**Background/Objectives:** Interleukin-1 (IL-1) is a pivotal mediator in the pathological progression of osteoarthritis (OA), playing a central role in disease progression. However, the rapid clearance of IL-1 receptor antagonist (IL-1Ra) from the joints may hinder the efficacy of intra-articular IL-1Ra injections in reducing OA-associated pain or cartilage degradation. **Methods:** Sustaining sufficient levels of IL-1Ra within the joints via adeno-associated virus (AAV)-mediated gene therapy presents a promising therapeutic strategy for OA. In this study, we constructed an IL-1Ra expression cassette employing intron insertion in the coding sequence (CDS) region to enhance protein expression levels. Furthermore, we incorporated precisely targeted liver-specific microRNA (miRNA) sequences to specifically downregulate transgene expression within hepatic tissues, thereby ensuring more targeted and controlled regulation of gene expression. **Results:** A rat model of OA was employed to compare the efficacy of AAV5 and AAV9 for IL-1Ra delivery at both high and low doses. It was observed that low-dose, but not high-dose, AAV9-IL-1Ra resulted in a significant reduction in joint swelling, accompanied by a decrease in the diameter of the affected area and the preservation of biomarkers associated with trabecular bone integrity. **Conclusions:** These results highlight the great potential of AAV9-IL-1Ra in osteoarthritis therapy, with the promise of achieving long-term improvement through a single intra-articular injection.

## 1. Introduction

Osteoarthritis (OA) represents the most prevalent form of arthritis and is a principal cause of disability among the elderly population on a global scale. In 2019, about 528 million people worldwide were living with osteoarthritis, an increase of 113% since 1990 [1]. Despite its prevalence, treating OA remains a significant challenge. Pharmacological treatments are predominantly associated with the relief of symptoms. Novel therapeutic approaches for OA are being sought within biologics and regenerative medicine. The rationale behind regenerative therapy in OA is based on the assumption that administered cells may engraft at the lesion site and differentiate into chondrocytes. However, recent studies have demonstrated that cells, particularly when injected in suspension, undergo apoptosis rapidly after exerting a transient paracrine effect [2]. The challenge of ensuring long-term cell survival remains unresolved in treating osteoarthritis using regenerative medicine. Most biologics developed to treat OA are anti-cytokine antibodies designed to reduce OA-related inflammation. This strategy has been successful in rheumatoid arthritis, a disease associated with more pronounced inflammation [3]. Notably, there are currently no therapeutic agents capable of halting the underlying disease processes [4].

In the past decade, significant advances have been made in understanding OA pathogenesis on cellular and molecular levels [5]. Furthermore, numerous natural proteins have been identified that show promise as disease-modifying agents. Interleukin (IL)-1 is one of the most well-characterized pro-inflammatory cytokines. IL-1 is synthesized locally by chondrocytes and synovial cells, acting as a central mediator of disease progression. It stimulates the production of inflammatory mediators, including prostaglandin E, nitric oxide synthase, chemokines, and other cytokines, within the joint microenvironment. Moreover, interleukin-1 directly promotes the expression of matrix metalloproteinases (MMPs) and other matrix-degrading enzymes that contribute to cartilage degeneration. Therefore, there have been extensive efforts to treat OA by inhibiting IL-1 [4,5,6]. The interleukin-1 receptor antagonist (IL-1Ra) is a naturally occurring substance that can effectively block IL-1 signaling [7]. However, the intra-articular injection of IL-1Ra was ineffective in reducing OA-associated pain or cartilage turnover during weeks of administration in a randomly controlled phase II clinical trial [8]. It is hypothesized that this may be related to the rapid clearance of IL-1Ra from the joints [9]. Maintaining a sufficient concentration of IL-1Ra at the site of the OA lesion may be an effective method of inhibiting the OA process.

Adeno-associated virus (AAV)-based gene therapy may offer an efficacious alternative. The AAV is regarded as the most promising vector for in vivo gene delivery, as it is mostly non-integrative and has high efficiency, long-term stability, and low toxicity [10]. It is characterized as a replication-deficient vector that cannot replicate autonomously, which leaves the target gene unintegrated into the chromosome, manifesting as an independent extrachromosomal DNA episome within the nucleus [10,11]. Recombinant AAV (rAAV) vectors demonstrated potential as a gene therapy delivery system for maintaining long-term, sustained expression of therapeutic proteins in ocular [12] and joint [13] applications based on results from preclinical studies. Although the AAV demonstrated a favorable safety profile in the vast majority of clinical trials, a small number of patients exhibited hepatotoxicity and experienced serious adverse effects. A total of four patient deaths were reported in the Audentes trial for X-linked myotubular myopathy, which may be attributed to hepatotoxicity due to AAV at high doses. These four patients received AAV gene therapy (AT132) at a dose of 1.3 × 10^14^ GC/kg (*n* = 1) or 3 × 10^14^ GC/kg (*n* = 3), exhibited evidence of hepatic dysfunction and ultimately died within 3–4 weeks [14]. Given that the liver is a primary target organ for AAV, it is imperative to consider methods of minimizing the potential impact on the liver when administering IL-1Ra using AAV to treat osteoarthritis (OA) via intra-articular injection [15,16].

In the present study, we designed an IL-1Ra expression cassette and enhanced transgene expression through the strategic insertion of introns into the coding sequence region. Furthermore, we inhibited transgene expression in the liver by incorporating liver-specific miRNA target sequences into the 3′-untranslated region (3′-UTR) of the aforementioned cassette. A comparison was conducted of the biodistribution and sustained expression of four AAV serotypes (AAV5, AAV6, AAV9, and AAVv128) following intra-articular injection in mice. The results demonstrated that AAV9 exhibited the highest and most sustained articular luminal expression. In a rat model of OA, a substantial reduction in joint swelling was observed, along with a decrease in the diameter of the affected area and the maintenance of biomarkers related to trabecular bone. These findings suggest that AAV-mediated gene therapy with IL-1Ra may hold considerable promise for treating OA.

## 2. Materials and Methods

### 2.1. Cell Lines

HEK293 cells, sourced from ATCC (ATCC catalog # CRL-3216, Gaithersburg, MD, USA), were maintained in a standardized environment using Dulbecco’s Modified Eagle Medium (DMEM; Gibco, #C11885500BT, Carlsbad, CA, USA) supplemented with 10% fetal bovine serum (Gibco, #A5256701, Carlsbad, CA, USA) and 1% penicillin–streptomycin antibiotic mixture (Gibco, #15140-122, Carlsbad, CA, USA) to ensure optimal growth conditions. Cells were cultured in a humidified incubator maintained at 37 °C with 5% CO_2_ for consistency. Prior to conducting in vitro expression assays utilizing various IL-1Ra cDNA constructs, cells were seeded in 6-well plates at a precise density of 3 × 10^5^ cells per well 18 to 24 h before transfection to ensure proper attachment and growth. Transfection was performed using Lipofectamine 2000 (Thermo Fisher Scientific, #246281, Waltham, MA, USA), adhering strictly to the manufacturer’s protocol for optimal efficiency. Following a 72-h incubation period post-transfection, cells were harvested, and the conditioned cell culture media were collected for subsequent analysis using enzyme-linked immunosorbent assay (ELISA, Abcam, #ab211650, Cambridge, UK) techniques to accurately quantify IL-1Ra expression levels.

### 2.2. Viral Vector Production

The recombinant adeno-associated viral (rAAV) vectors were produced employing a tripartite plasmid transfection approach [17]. HEK293 cells served as the host packaging cell line. This process involved the utilization of the pHelper plasmid alongside pRC plasmids encoding for AAV2 Rep and either AAV5, 6, 9, or AAV.v128 Cap proteins. Additionally, cis-plasmids (transgene plasmids) harboring IL-1Ra or reporter genes (luciferase, eGFP, and mCherry) under the transcriptional control of both the chicken beta-actin (CB) and U1a promoters were incorporated. The insertion of introns into the coding sequence (CDS) region of IL-1Ra adhered strictly to the principles governing intron biology, as detailed in Supplementary Material S1.

Prior to transfection, cells were seeded onto 150 mm plates and incubated overnight. Subsequently, a 1:1:1 molar ratio of pHelper, pRC, and cis-plasmids was co-transfected into the cells. Then, 72 h post-transfection, cells were harvested via centrifugation at 2000× *g* at 4 °C for 15 min. The resultant cell pellet was resuspended in a lysis buffer composed of 150 mM NaCl, 1 mM MgCl_2_, and 50 mM Tris-HCl (pH 8.0) and subjected to three freeze–thaw cycles to release the rAAV particles. To eliminate unpackaged rAAV DNA, the crude lysate was treated with Benzonase and 10% deoxycholic acid at 37 °C for 1 h. Following centrifugation at 12,000× *g* for 30 min to remove cellular debris, the rAAV vectors were purified via affinity chromatography using POROS™ CaptureSelect™ AAVX Affinity Resin (Thermo Fisher Scientific, #A36745, Waltham, MA, USA), followed by anion exchange chromatography. The vector titers (vg/mL) were accurately determined via droplet digital PCR (ddPCR). Crystal Reader software (version, 3.6.1.3) was then opened to collect and process the PCR data. The IL-1Ra primers are as follows: Forward, 5′-CTCTCTGTTCACAGGCCCAG-3′, Reverse, 5′-CGCCGTGAATTCCCAGAAAC-3′, Probe, 5′-FAM-ACAATCAGCTGGTGGCCGGC-BHQ1-3′.

### 2.3. Animals

Female SD rats and Balb/c mice, aged precisely 4 to 5 weeks, were procured from Sichuan Vital River Laboratory Animal Technology Co., Ltd. (Chengdu, China) and subsequently randomized into experimental groups. The animals possessed a valid Experimental Animal Production License: SCXK (Sichuan) 2023-0040, and their quality was confirmed by Experimental Animal Quality Certificate No. 511215600000269. All animal husbandry practices and experimental procedures were executed at the Beijing Match Medicine Research Institute Co., Ltd., (Beijing, China) adhering strictly to the guidelines approved by the Institutional Animal Care and Use Committee (IACUC) of the Institute. The animals were maintained in standardized environmental conditions, including a temperature-controlled range of 20 °C to 25 °C, humidity maintained between 40% and 70%, and a 12-h light/dark cycle to mimic diurnal rhythms. Access to food and water was provided ad libitum to ensure the optimal health and well-being of the experimental subjects.

### 2.4. In Vivo Bioluminescence Imaging

Mice underwent intra-articular injection of AAV-luciferase into the ankle joint at a precise dosage of 1 × 10^11^ vg per joint. Prior to bioluminescence imaging, the animals were thoroughly anesthetized using a combination of ketamine (0.1 mg/g) and xylazine (0.02 mg/g) mixed with sterile water in the ratio of 0.6:1:8.4, respectively. For bioluminescence visualization, each mouse received an intraperitoneal injection of 200 μL of D-Luciferin (Thermo Fisher Scientific, #L2916, Waltham, MA, USA) at a dose of 150 mg/kg. Then, 10 min post-injection, while the animals remained under anesthesia, bioluminescence images were acquired using a luminescence imager (Xenogen, IVIS Imaging System 200). The in vivo bioluminescence imaging protocol was systematically executed from day 33 to day 166 following the administration of the viral vector.

### 2.5. Rat OA Model

Prior to surgical modeling, rats underwent an overnight fast and were subjected to surgery the subsequent day. Following anesthesia induction with 5% isoflurane, the right knee joint of each rat was meticulously prepared by shaving, cleaning, and disinfecting the area. A precise incision was made medial to the patellar ligament, allowing for sequential dissection of the skin, subcutaneous tissue, and joint capsule. The patella was then carefully dislocated laterally to expose the knee joint. Using a scalpel, the anterior cruciate ligament was horizontally transected, and a subsequent 45-degree outward rotation from its tibial origin facilitated the removal of one-third of the medial meniscus. Following these manipulations, the patella was repositioned, hemostasis was ensured, and the skin was meticulously sutured.

After the modeling process was completed for two weeks, Researcher A systematically randomized the entire cohort of 60 female rats into six distinct groups: sham surgery, vehicle control (model), high-dose AAV5, low-dose AAV5, high-dose AAV9, and low-dose AAV9. Each group was then assigned a unique identifier, ranging from G1 to G6. Subsequently, Researcher B assigned labels P1 through P6 to the test drugs. Researcher C, who remained blinded to the GX and PX labeling details, was responsible for administering the intra-articular injections and performing statistical analyses on all pertinent research indicators that followed. Ultimately, Researchers A and B jointly conducted the unblinding procedure. Two weeks post-modeling, intra-articular injections were administered to the surgically manipulated knee joints of the rats. In contrast, the sham surgery group underwent identical surgical procedures up to the point of patella dislocation, with no transection of the anterior cruciate ligament or removal of the meniscus. AAV vectors were administered in a standardized volume of 40 μL via direct intra-articular injection into the right knee joint. Rats were subjected to daily monitoring, and the clinical score of each animal was rigorously determined according to previously established and validated criteria [18]. The primary indicators of assessment encompassed the presence of erythema, as well as the degree and extent of swelling, ensuring a comprehensive and scientific evaluation of the experimental outcomes.

### 2.6. X-Ray Clinical Score

The DR clinical score was evaluated. Specifically, each rat underwent X-ray imaging using the Digital Veterinary X-Ray System (VDR-1800, manufactured by Suzhou VetRay Medical Equipment Co., Ltd., Suzhou, China). The radiological analysis was methodically conducted, focusing on joint swelling, bone erosion, and joint space dimensions. This analysis utilized the well-established Kellgren–Lawrence scoring system [19], ranging from 0 to IV, with the following definitions:

- 0: A normal state, with no observed narrowing of the joint space and the absence of reactive bone changes on the X-ray;
- I: There is a possibility of mild narrowing of the joint space and initial, minor osteophyte formation;
- II: The definite presence of small osteophytes and potential narrowing of the joint space, as evidenced on the X-ray;
- III: Characterized by multiple, moderate-sized osteophytes, definitive narrowing of the joint space, some degree of subchondral bone sclerosis, and a likelihood of bony deformity within the joint;
- IV: A severe state with numerous large osteophytes, marked narrowing of the joint space, pronounced subchondral bone sclerosis, and overt bony deformity of the joint.

The radiological assessments were assigned scores by independent, blinded observers who were uninformed of the treatment conditions to ensure objectivity. The assessment timeline encompassed one evaluation prior to drug administration, followed by evaluations at 4 and 8 weeks post-administration, totaling three assessments throughout the study period.

### 2.7. Micro-CT Examination

Upon euthanizing the rats, the right-side joints were carefully isolated, and a micro-CT analysis was conducted to examine the joints of animals within each experimental group. The detailed detection procedures are outlined below:

(1) μCT Scanning Procedure: Prior to Micro-CT scanning, all sampled specimens underwent rigorous fixation processing. The scanning parameters were precisely calibrated to ensure accuracy: a tube current of 200 μA, a voltage of 85 KV, a scanning resolution of 10.154648 μm across the entire object, an exposure time of 384 ms, and a scanning angle of 180 degrees. To ensure consistency, a phantom was scanned under identical conditions for calibration purposes. Subsequently, the raw images were acquired. (2) μCT Reconstruction Process: Utilizing the advanced three-dimensional reconstruction software NRecon (version V1.7.4.2, Bruker, Germany), the raw images were reconstructed for predefined regions of interest. Prior to reconstruction, a preliminary review of the reconstructed image was performed. To optimize image quality and minimize artifacts, the reconstruction parameters were meticulously adjusted: Smoothing was set to 5 for enhanced smoothness, the beam-hardening correction was adjusted to 8 to account for beam intensity variations, and ring artifacts reduction was set to 25% to mitigate circular artifacts. Once the parameters were finalized, the specified folder was configured to initiate the image reconstruction process. (3) μCT Analysis Methodology: The Region of Interest (ROI) was thoroughly analyzed using CT Analyser software (version 1.20.3.0, Bruker, Germany). Uniform parameters were applied across all samples, enabling the software to accurately calculate key parameters such as the Total Volume (TV) of tissue, Bone Volume (BV), Volume Ratio (BV/TV), Bone Surface (BS), Trabecular Number (Tb.N), Trabecular Thickness (Tb.th), Trabecular Separation (Tb.Sp) and ensure consistency in the assessment of bone structure characteristics.

### 2.8. ELISA Assay

Upon collection, the ankle joint tissues, liver, and serum samples from animals were promptly snap-frozen in liquid nitrogen to preserve their integrity. Following homogenization of the tissues, centrifugation was performed, and the resulting pellet was discarded to isolate the supernatant. Subsequently, a standardized Human IL-1Ra quantification kit (Abcam, catalog #: ab211650, Cambridge, UK) was employed to accurately measure the expression levels of IL-1Ra within the supernatant, ensuring scientific rigor and precision in the analysis.

### 2.9. Statistical Analysis

The quantitative data are comprehensively presented as the mean value accompanied by the standard error of the mean (SEM) to ensure precision and reproducibility. The visualization of these data was facilitated using GraphPad Prism 10.0 software. For datasets that adhere to a normal distribution and exhibit homogeneity of variance, statistical significance was determined by applying a one-way analysis of variance (ANOVA). Conversely, for data that deviate from a normal distribution or show heterogeneity of variance, the Kruskal–Wallis H test was employed for statistical analysis. Further, to compare differences between specific groups, the Mann–Whitney U test (M-W test) was utilized. Statistical significance was established at a threshold of *p* ≤ 0.05, ensuring rigor and objectivity in interpreting results.

## 3. Results

### 3.1. Optimize the Expression Cassette of IL-1Ra Plasmid Using Intron Insertion into CDS Technology

**Figure 1:**
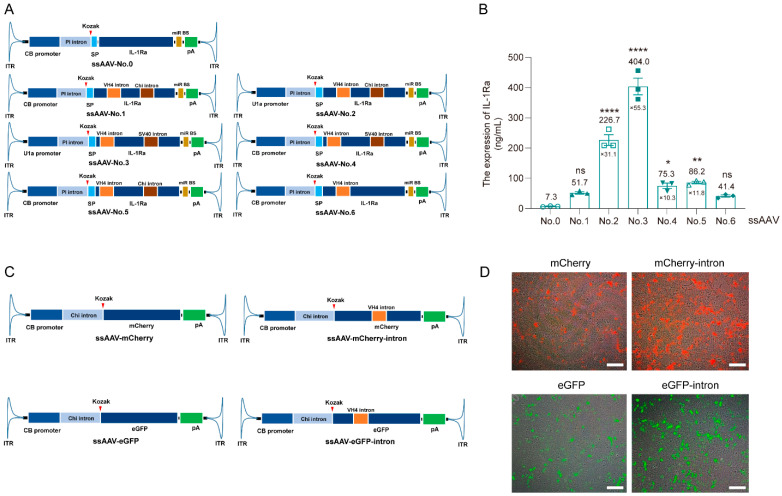
The construction of plasmid cassettes with intron insertion in the transgene CDS region. Note: (**A**), a schematic illustration detailing the construction of multiple plasmid cassettes, each featuring an intron insertion specifically within the coding sequence (CDS) of the IL-1Ra gene. (**B**), the quantification of the protein expression levels of IL-1Ra plasmids, achieved via ELISA analysis subsequent to transduction into HEK293 cells (*n* = 3). (**C**), a conceptual diagram outlining the design and construction of plasmid cassettes, which incorporate the VH4 intron within the CDS of reporter genes, specifically eGFP and mCherry. (**D**), the visualization and verification of fluorescent signals emitted by eGFP-intron and mCherry-intron plasmids, utilizing an inverted fluorescence microscope, after these constructs have been transduced into and expressed by HEK293 cells. Compared with No. 0, ns means not significant (*p* > 0.05), * *p* < 0.05, ** *p* < 0.01, **** *p* < 0.0001; scale bar: 50 μL.

We constructed various plasmid expression cassettes, each featuring IL-1Ra as the transgene, by strategically combining various promoters and introns. Furthermore, we implemented an innovative approach involving the insertion of 1–2 introns within the coding sequence (CDS) domain of the transgene to augment protein expression levels (Figure 1A). Quantitative analysis via an enzyme-linked immunosorbent assay (ELISA) revealed a significant elevation in protein expression levels for constructs No. 5 and No. 6 compared with No. 0, with increases from 7.3 ng/mL to 86.2 ng/mL (11.8-fold) and from 7.3 ng/mL to 41.4 ng/mL, respectively (Figure 1B, *p* < 0.01). This underscores the effectiveness of our intron insertion strategy in improving transgene protein expression. Notably, although variations in intron insertion sites within the CDS region exhibited modest impacts on expression levels, these differences did not attain statistical significance (e.g., No. 1 vs. No. 5, 51.7 ng/mL vs. 86.2 ng/mL). Additionally, the choice of intron did not significantly alter expression levels when driven by the CB promoter (No. 4 vs. No. 5, 75.3 ng/mL vs. 86.2 ng/mL). Intriguingly, the U1a promoter was found to significantly outperform the CB promoter in driving IL-1Ra protein expression (No. 1 vs. No. 2, 51.7 ng/mL vs. 226.7 ng/mL) (Figure 1B). Consequently, based on its superior expression level, we selected No. 3 as the optimal plasmid vector for subsequent experimental investigations (Figure 1B).

To rigorously evaluate the universal applicability of our intron insertion strategy within the CDS region of the transgene to enhance protein expression, we extended our experiments to include two reporter genes, eGFP and mCherry, by incorporating the VH4 intron into their respective sequences (Figure 1C). As evident in Figure 1D, the fluorescence intensities of the modified eGFP-intron and mCherry-intron plasmids, both containing introns, were significantly improved compared to their non-intron-containing counterparts (Figure 1D). This comprehensive validation, spanning IL-1Ra, eGFP, and mCherry, underscores the robustness and feasibility of our strategy to enhance protein expression through intron insertion within the CDS region of the transgene.

### 3.2. Intra-Articular Injection of AAV9-Luciferase-miR122 BS Vector Significantly Reduces the Expression Level in Liver

**Figure 2:**
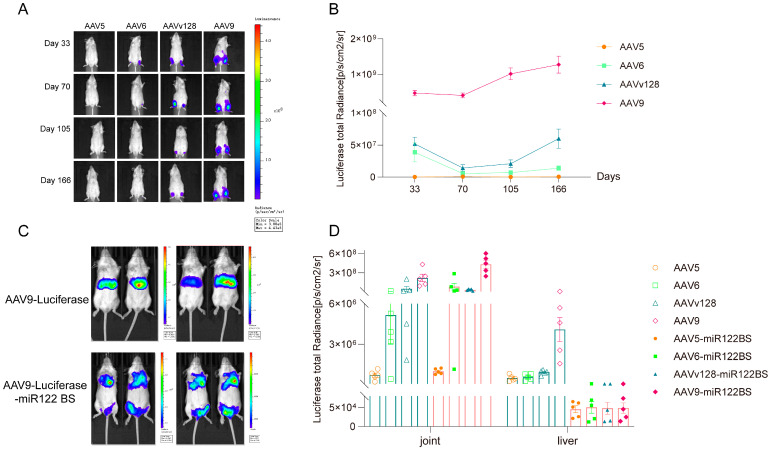
In vivo bioluminescence imaging detection of the expression levels of different rAAV serotypes in mice. Note: (**A**), longitudinal in vivo bioluminescence imaging to track expression dynamics post-intra-articular administration into mice (*n* = 5, 1 × 10^11^ vg/joint); (**B**), the quantitative assessment of long-term expression levels within the joint cavities of mice; (**C**), in vivo bioluminescence imaging of the distribution of AAV9-luciferase and AAV9-luciferase-miR122 BS constructs following intravenous injection (*n* = 4, 1 × 10^12^ vg/mice); (**D**), comparative in vivo fluorescence analysis of the expression levels achieved through intra-articular injection of various AAV serotypes (AAV5, AAV6, AAVv128, and AAV9), both with and without the inclusion of miR122 BS, to evaluate the impact of serotype and miRNA targeting on liver-specific transgene expression (*n* = 5, 1 × 10^11^ vg/joint).

We opted for the AAVv128 capsid, renowned for its superior transduction efficiency compared to the AAV8 [20,21], alongside AAV5, AAV6, and AAV9, to systematically evaluate their transduction efficiencies following intra-articular administration. In vivo imaging analysis revealed that all four serotypes maintained consistent expression levels within the joint cavity for 166 days, with AAV9 exhibiting peak transduction efficiency (Figure 2A,B).

It is a well-established fact that all AAV serotypes display a strong tropism toward the liver. This phenomenon was reconfirmed through intravenous delivery of the AAV9-luciferase vector, as evidenced in Figure 2C. Given the abundant expression of miR122 in the liver, incorporating a miR122 binding site (BS) into the AAV construct has been shown to effectively diminish AAV-mediated transgene expression in the liver, thereby enhancing overall safety. We achieved this by strategically positioning the miR122 BS at the 3′ end of the transgene’s coding sequence (CDS) and employing a triple-plasmid transient transfection approach to generate the AAV9-luciferase-miR122 BS vector. Notably, post-intravenous injection of this modified vector (Figure 2C) demonstrates a markedly attenuated fluorescent signal of luciferase in the liver, indicating a substantial reduction in AAV expression mediated by miR122 BS (Figure 2C).

To further explore the impact of miR122 BS, we conducted a comparative analysis by administering AAV5, AAV6, AAVv128, and AAV9 into the joint cavity. The results presented in Figure 2D underscore the superiority of AAV9 in achieving the highest transduction efficiency within the joint post-intra-articular injection, albeit accompanied by the highest liver transduction efficiency (Figure 2D). Surprisingly, it is worth noting that the strategic integration of miR122 BS into AAV9-luciferase significantly diminished its expression in the liver by approximately 100-fold (4.5 × 10^6^ vs. 5 × 10^4^), offering a promising strategy to enhance the safety profile of AAV intra-articular injection and mitigate the risk of off-target effects (Figure 2D).

### 3.3. Low-Dose AAV Has Good Efficacy on OA Model in Rats, While High-Dose AAV Has Poor Therapeutic Effect

**Figure 3:**
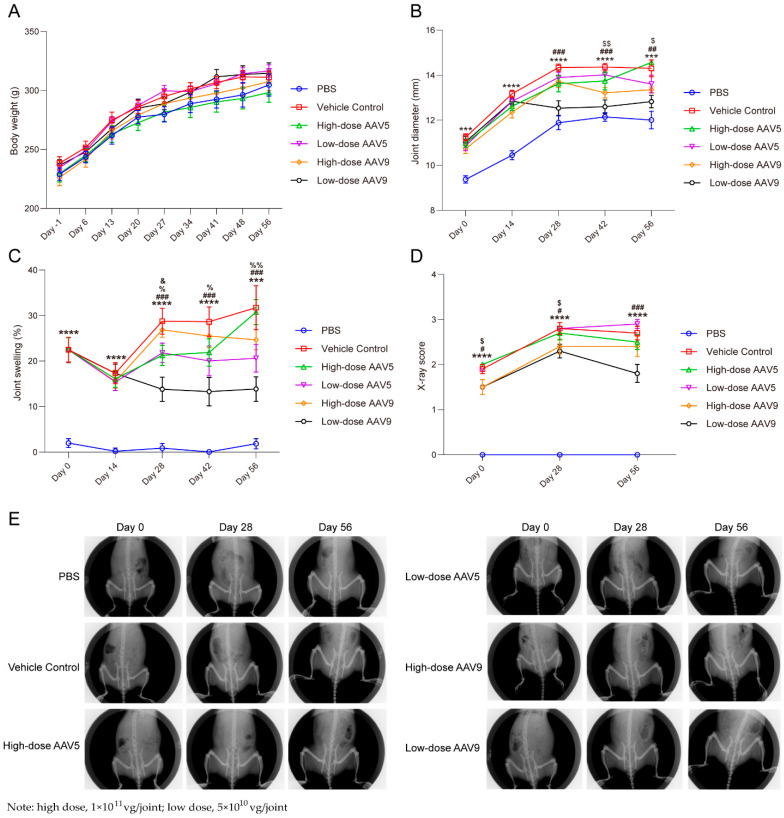
The low-dose AAV-IL1Ra vector exhibits good pharmacodynamic effects in a surgically induced OA rat model. Note: (**A**), body weight (g); (**B**), joint diameter (mm); (**C**), joint swelling (%); (**D**), X-ray score; (**E**), representative X-ray images of various dose groups in the OA rat model. Low dose: 5 × 10^10^ vg/joint; high dose: 1 × 10^11^ vg/joint; *n* = 10. Compared with PBS, *** *p* < 0.001, **** *p* < 0.0001 (vehicle group); compared with the vehicle group, ^#^ *p* < 0.05, ^##^ *p* < 0.01, ^###^ *p* < 0.001 (low-dose AAV9 group); compared with the vehicle group, ^$^ *p* < 0.05, ^$$^ *p* < 0.01 (high-dose AAV9 group); compared with the vehicle group, ^%^ *p* < 0.05, ^%%^ *p* < 0.01 (low-dose AAV5 group); compared with the vehicle group, ^&^ *p* < 0.05 (high-dose AAV5 group).

We developed a robust and reproducible osteoarthritis (OA) model in rats by surgically transecting the anterior cruciate ligament and partially excising the meniscus in the right knee joint. Subsequently, we rigorously evaluated the pharmacodynamic impacts of the AAV-No. 3 vector on OA progression in these rats. Our experimental design entailed modeling the rats, administering AAV two weeks thereafter (designated as D0), and then systematically monitoring pertinent indicators at biweekly intervals. As evident in Figure 3B, the vehicle control group exhibited a marked increase in swelling at all monitored time points compared to the PBS group (*p* < 0.001), validating the successful induction of OA through our surgical protocol. During the initial two weeks post-AAV administration, no reduction in swelling was observed in any of the AAV-treated groups, plausibly due to the AAV’s expression kinetics, which typically peak between 21 and 28 days, with IL-1Ra protein levels insufficient for a therapeutic effect within this early period.

Remarkably, after 28 days, the low-dose AAV9 group (5 × 10^10^ vg/joint; *n* = 10) demonstrated significant reductions in swelling on days 28, 42, and 56 compared to the vehicle control, whereas the high-dose AAV9 group (1 × 10^11^ vg/joint; *n* = 10) exhibited significant reductions only on days 42 and 56, albeit with inferior efficacy. This disparity may stem from the immunogenicity of the AAV9 capsid at higher doses, compromising therapeutic outcomes. In contrast, both high- and low-dose AAV5 groups failed to significantly mitigate swelling compared to the vehicle control (5 × 10^10^ vg/joint; high dose: 1 × 10^11^ vg/joint; *n* = 10). Intriguingly, the low-dose AAV5 group displayed modest therapeutic trends (data presented). In contrast, the high-dose AAV5 group exacerbated swelling, suggesting that the high-dose AAV5 capsid may have induced a cellular immune response targeted against it within the intra-articular area, subsequently eliciting a more robust inflammatory reaction and heightened swelling (Supplementary Tables S1 and S2).

Concordant findings were observed in joint swelling diameter measurements, with high-dose AAV5 and AAV9 groups exhibiting significantly greater diameters than their respective low-dose counterparts. Notably, the low-dose AAV9 group showed a significant decrease in swelling diameter versus the vehicle control, indicating superior therapeutic potential. X-ray scoring further corroborated these findings, revealing a significant reduction in the low-dose AAV9 group’s X-ray score compared to the vehicle control (Figure 3D,E, *p* < 0.001). In contrast, no significant differences were noted in other groups on day 56. Collectively, these findings underscore the pivotal role of AAV dosage in modulating its therapeutic efficacy for intra-articular OA treatment. In the context of immune-mediated diseases, meticulous exploration of AAV dosage is imperative, given the non-negligible immune responses elicited by the AAV capsid itself.

### 3.4. AAV-IL-1Ra Gene Therapy Substantially Protects Trabecular Bone-Related Indicators in Animal Models

**Figure 4:**
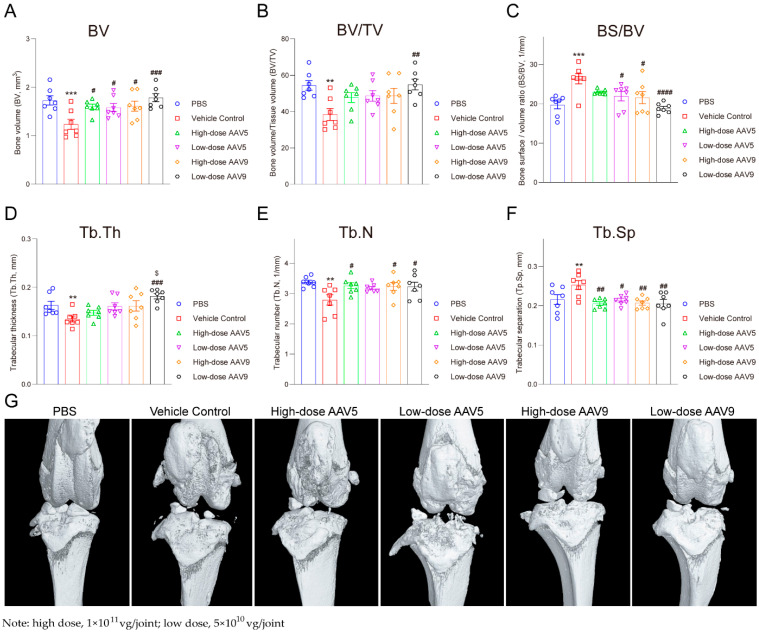
AAV-IL-1Ra gene therapy substantially protects trabecular bone-related indicators in animal models. Note: We evaluated the therapeutic effects of each group on the rat OA model using micro-CT analysis. (**A**), Bone Volume (BV); (**B**), Bone Volume/Tissue Volume (BV/TV); (**C**), Bone Surface/Volume Ratio (BS/BV); (**D**), Trabecular Thickness (Tb.Th); (**E**), Trabecular Number (Tb.N); (**F**), Trabecular Separation (Tb.Sp); (**G**), Representative Micro-CT images of various dose groups in the OA rat model. Low dose: 5 × 10^10^ vg/joint; high dose: 1 × 10^11^ vg/joint; *n* = 7; compared with PBS, ** *p* < 0.01, *** *p* < 0.001 (vehicle group); compared with the vehicle group, ^#^ *p* < 0.05, ^##^ *p* < 0.01, ^###^ *p* < 0.001, ^####^ *p* < 0.0001; compared with high-dose AAV5, ^$^ *p* < 0.05.

To assess the protective impacts of AAV-IL-1Ra vector on key trabecular bone parameters, we conducted Micro-CT analysis, focusing on Bone Volume (BV), Bone Volume/Tissue Volume (BV/TV), Bone Surface/Volume Ratio (BS/BV), Trabecular Thickness (Tb.Th), Trabecular Number (Tb.N), and Trabecular Separation (Tb.Sp). Our findings revealed that the low-dose AAV9 group (5 × 10^10^ vg/joint; *n* = 7) significantly enhanced BV and BV/TV compared to the vehicle control (Figure 4A,B, *p* < 0.01), while other groups displayed modest, non-significant protective trends. For BS/BV, the low-dose AAV9 group had a markedly reduced ratio (Figure 4C, *p* < 0.0001), with the low-dose AAV5 and high-dose AAV9 groups also demonstrating reductions (Figure 4C, *p* < 0.05), albeit not significantly different from the vehicle control (Figure 4C, 5 × 10^10^ vg/joint; high dose: 1 × 10^11^ vg/joint; *n* = 7).

Regarding Tb.Th, only the low-dose AAV9 group significantly augmented Trabecular Thickness in rats, approaching levels observed in the sham-operated group (Figure 4D, *p* < 0.001), whereas other AAV groups showed no therapeutic benefit. In particular, compared with high-dose AAV5, low-dose AAV9 still significantly enhances the efficiency of Tb.Th protection (Figure 4D, *p* < 0.05). In contrast, both high/low-dose AAV9 and high-dose AAV5 groups had significantly elevated Tb.N (Figure 4E, *p* < 0.05), matching sham-operated levels. Additionally, the low-dose AAV9 group and all AAV groups had significantly diminished Tb.Sp compared to the vehicle control (Figure 4F, *p* < 0.01). Micro-CT imagery underscored the low-dose AAV9 group’s superior therapeutic outcome (Figure 4G). Similar outcomes were observed in Safranin O/Fast Green and Hematoxylin-Eosin staining (Supplementary Figures S1 and S2). The low-dose AAV9 group demonstrated superior chondrocyte protection compared to the high-dose groups, which was consistent with the joint swelling results. The amount and morphology of articular cartilage in the low-dose AAV9 group were comparable to that of the negative control group (PBS), and no significant inflammation was observed. In conclusion, within the rat OA model induced by a meniscus tear, the application of a low-dose AAV9-IL-1Ra vector emerged as a relatively effective therapeutic approach for OA.

## 4. Discussion

Introns, being non-coding sequences inherent to eukaryotic genes, undergo excision during the post-transcriptional processing of mRNA and consequently do not contribute to the final protein coding sequence [22]. Nonetheless, contemporary research has unveiled that the strategic insertion of introns into the coding sequence (CDS) region can significantly modulate gene expression levels [23]. This manipulation primarily involves two pivotal aspects [23,24,25]: (1) The careful selection of introns tailored to the target gene’s unique characteristics and expression needs, prioritizing those with defined functional or regulatory properties. (2) The precise determination of the insertion site within the CDS, as its location critically impacts gene expression. The optimal insertion site is chosen, considering both the target gene’s structural and functional attributes, as well as the regulatory mechanisms of the chosen introns. Literature has documented that, upon transfection into IPSG cells, plasmids containing an intron insertion exhibited a 5-fold elevation in protein expression levels compared to those lacking an intron. Furthermore, a notable augmentation in fluorescence intensity was observed in mouse brain tissue sections [23]. Additionally, another study reported an 8.4-fold increase in protein expression for plasmids with intron insertions relative to those without when transfected into *Chlamydomonas reinhardt* cells [24].

Our findings (Figure 1A,B) reveal that the introduction of a single VH4 intron into the CDS of IL-1Ra resulted in a substantial increase in expression, from 7.3 ng/mL to 41.4 ng/mL (No. 0 vs. No. 6). Furthermore, the co-insertion of two introns, VH4 and Chi, led to an even more pronounced elevation in expression, reaching 51.7 ng/mL (No. 0 vs. No. 1). Notably, variation in expression levels was observed when the same two introns were inserted at different sites (No. 1 vs. No. 5, 51.7 ng/mL vs. 86.2 ng/mL), suggesting that the insertion site is a pivotal factor. In contrast, when two distinct introns were placed at the same site (Chi or SV40 intron), the difference in expression was minimal (No. 4 vs. No. 5, 75.3 ng/mL vs. 86.2 ng/mL), underscoring the role of intron identity in this context. These outcomes conclusively demonstrate that introns can potently enhance target gene expression, with the insertion site being a crucial determinant of protein expression levels.

Additionally, our study highlights that utilizing distinct promoters to initiate the expression of the same target gene can lead to substantial variations in expression levels (No. 1 vs. No. 2, 51.7 ng/mL vs. 226.7 ng/mL). In general, the choice of promoter significantly influences the level of expression of the target gene. Adopting a high-expression promoter facilitates the attainment of an effective therapeutic concentration of the target protein drug. Conversely, low-expression promoters often fail to achieve therapeutically effective concentrations in vivo, rendering the treatment ineffective. Even if an effective therapeutic concentration is achieved with a low-expression promoter, it typically necessitates a higher dose of rAAV administration. High doses of rAAV are more prone to induce adverse effects, including hepatotoxicity and inflammatory reactions. Consequently, opting for a high-expression promoter not only ensures therapeutic efficacy but also minimizes the dosing amount, thereby mitigating the potential treatment risks associated with rAAV administration. To further validate the robustness of our intron insertion approach, we extended our investigation to the reporter genes eGFP and mCherry, successfully demonstrating its versatility in enhancing protein expression through intron insertion. This strategy presents a promising avenue for enhancing protein expression efficiencies and potentially reducing production costs, as evidenced by our results (Figure 1C,D).

IL-1Ra, an antagonist to IL-1 (interleukin-1), operates primarily through competitive binding to IL-1 receptors, effectively inhibiting the biological activity of IL-1. Under physiological conditions, a delicate balance exists between IL-1 and IL-1Ra, which is indispensable for maintaining normal joint physiological functions. Nevertheless, the overexpression of IL-1Ra can disrupt normal immune regulation, leading to immune dysregulation. This immune imbalance may exacerbate rheumatoid arthritis (RA) symptoms, trigger novel autoimmune responses, enhance susceptibility to specific pathogens, and potentially initiate or perpetuate chronic inflammatory states, ultimately resulting in tissue damage and functional impairment [7,26]. When AAVs are administered either systemically or locally, a fraction of them continue to express in the liver, particularly if the transgene encodes an inflammation-related factor, potentially inducing hepatotoxicity (Figure 2C). To mitigate this risk, we incorporated miR122 binding sites (BS) into the plasmid expression cassette, thereby reducing AAV expression in the liver and subsequently decreasing the likelihood of hepatotoxicity (Figure 2D).

miR122 is a microRNA that exhibits remarkable liver specificity, with its expression levels in liver tissues substantially higher than in any other tissue [27,28,29]. This distinctive feature renders miR122 a pivotal molecular instrument for modulating liver gene expression [30]. The deliberate incorporation of the miR122 binding site (miR122 BS), the target sequence recognized by miR122, into AAV vectors specifically targets the 3′-untranslated region (3′-UTR) of the transgene mRNA carried by the vector [15,31]. This targeted interaction between miR122 and the 3′-UTR results in either degradation or translational suppression of the transgene mRNA, thereby significantly reducing its expression levels, specifically within liver tissues [32].

Many experimental studies have conclusively shown that integrating miR122 BS into AAV vectors effectively mitigates transgene expression in liver tissues [32,33,34,35]. Notably, research conducted by Guo et al. highlighted that fusing miR122 BS to the 3′-UTR of AAV9-Tnnt2 vectors drastically mitigated transgene leakage in liver tissues while enhancing its cardiac tissue specificity. This refined AAV vector exhibited remarkable therapeutic potential in myocardial infarction gene therapy, accomplishing its therapeutic goals without inducing any collateral liver damage [16]. Our findings, as depicted in Figure 2C,D, underscore the capacity of miR122 BS-modified AAV9 vectors to nearly abrogate transgene expression in the liver by a factor of 100 subsequent to intra-articular injection. On the other hand, long-term transgene expression is the major advantage of AAV vectors, which makes AAV vectors the optimal in vivo gene delivery tools. The administration of anti-miR-122 “Tough Decoys” via AAV resulted in a serum cholesterol reduction of >30% in mice that persisted over 25 weeks [36]. The expression of miR122 BS through AAV could sustainably reduce transgene protein levels in the liver.

In essence, the potency of miR122 BS in curbing AAV expression in the liver stems from three pillars: miR122’s exclusive expression in liver tissues, its inherent gene silencing mechanism, and its aptitude for refining AAV vector tissue specificity. These defining characteristics confer upon miR122 BS significant application potential in gene therapy, bolstering specificity, propelling technological advancements, and fostering scientific progress.

AAV is an attractive option for in vivo gene delivery, characterized by its non-integrative nature and high efficiency, long-term stability, and low toxicity [10]. Notably, AAV is the only gene therapy vector classified by the NIH as RG1 (the highest level of safety) and free of potential pathogenicity [37]. AAV has risen to prominence among viral vectors due to its exceptional capacity to ensure durable in vivo expression spanning decades with a solitary injection [10,38]. Our ongoing studies have tracked AAV expression in mouse joint cavities for up to 166 days post-intra-articular injection. AAV has demonstrated promising long-term efficacy and safety in treating other disease areas, including neovascular age-related macular degeneration (nAMD). RGX-314 and KH631 are both AAV-anti-VEGF gene therapy products for treating nAMD. Their sustained efficacy has been demonstrated over 2 years [39] and 96 weeks [12] in patients and rhesus monkeys, respectively, and they have a favorable long-term safety profile. In addition, the successful clinical outcomes of AAV-based gene therapies [10,40], including Glybera, Luxturna, Zolgensma, Upstaza, Roctavian, Hemgenix, and Elevidys, have firmly established the safety and efficacy of AAV vectors. Thereby, we consider low-dose AAV9-IL-1Ra to be a prospective treatment for osteoarthritis. Longer-term non-human primate and clinical trials will probably be conducted after further refinement of the basic research.

Local joint administration is a promising gene delivery approach for OA, as it enables the achievement of effective therapeutic concentrations at the lesion site while mitigating systemic side effects. Currently, the animal models commonly employed for studying osteoarthritis encompass mice, rats, rabbits, dogs, sheep, horses, and non-human primates. However, when considering factors such as experimental costs, animal ethics, and duration, small-animal models are preferentially selected for assessing in vivo efficacy during the drug development process. Rats, in particular, offer several advantages over mice, including ease of surgical sampling and intra-articular manipulations, joint load-bearing characteristics that are more similar to humans, thicker cartilage, more complex joint structures, and the capacity to surgically replicate partial or complete osteoarthritis-related cartilage damage [41,42]. Consequently, in the initial stages of drug development, the rat OA model stands as the most frequently utilized model for simulating osteoarthritis. Consequently, in the initial stages of drug development, the rat OA model is the most frequently utilized model for simulating osteoarthritis. However, achieving high transduction efficiency remains a formidable challenge in intra-articular gene therapy. AAV6 and AAV9 exhibit high transduction efficiency in muscle and cardiomyocytes, rendering them suitable for gene therapy targeting muscle-related diseases [20,43,44]. In contrast, AAV5, despite demonstrating lower transduction capability in muscle cells compared to AAV6 and AAV9, boasts the lowest relative levels of immune-neutralizing antibodies and enhanced safety, thereby meriting inclusion in our investigation [45]. Additionally, our previous research has shown that AAVv128 possesses significantly superior transduction ability compared to AAV8 [20,21]. Specifically, following intra-articular injection, AAVv128 effectively transduces synovial cells and exhibits therapeutic potential for rheumatoid arthritis, making it another candidate for our investigation [20]. In the present study, we conducted a comparative analysis of the transduction efficiency of various AAV capsids within the joint cavity, revealing that the AAV9 capsid exhibited superior transduction efficiency compared to AAV6, AAV5, and AAVv128. Notably, we demonstrate that miR122 binding site (BS)-modified AAV9 vectors significantly diminish transgene expression in the liver by approximately 100-fold following intra-articular injection, highlighting their potential for reducing off-target effects (Figure 2C). Consequently, we affirm the promising safety profile and therapeutic potential of AAV9-IL-1Ra-miR122 BS vectors as a gene therapy for OA.

AAV-mediated gene therapy has emerged as a promising avenue for treating osteoarthritis (OA), as evidenced by studies utilizing AAV2.5 vectors to deliver IL-1Ra, resulting in favorable efficacy outcomes [13,46,47]. The rat OA model employed in this research, established through surgical transection of the anterior cruciate ligament and partial meniscectomy in the right knee joint, closely mimics human OA pathology, enabling robust and reproducible outcomes [48]. Following induction, untreated rats exhibited hallmark OA features, including joint swelling, tissue damage, and decreased trabecular bone-related markers.

The administration of a low dose of 5 × 10^10^ vg/joint of AAV prior to disease onset effectively suppressed inflammatory responses, alleviating swelling and pathological changes (Figure 3 and Figure 4). This was attributed to the sustained expression capability of AAV, obviating the need for repeated dosing. However, higher doses (1 × 10^11^ vg/joint), while partially alleviating swelling, raised concerns about inducing an immune response that could exacerbate joint inflammation and undermine therapeutic gains (Figure 3 and Figure 4). Notably, post-disease administration of the vector showed reduced efficacy due to pre-existing pathological damage, emphasizing the criticality of timely intervention. The administration of high-dose rAAV may induce transgene overexpression in target tissues, potentially triggering an immunotoxicity response. Specifically, an excessive transgene containing unmethylated CpG motifs, akin to those found in microbial DNA, can be recognized and activated by Toll-like receptor 9 (TLR-9) within the endosomal area [49]. The activation of TLR-9 in rAAV not only results in the acute expression of cytokines such as interleukin-6 (IL-6) and tumor necrosis factor-alpha (TNF-α) downstream of TLR signaling but also promotes the infiltration of cytotoxic CD8+ T cells, ultimately leading to the loss of transduced target cells [50,51]. This, consequently, diminishes the therapeutic effect. Additionally, when rAAV carries a foreign transgene within the endogenous copy, the transgene product may be recognized as a non-self-antigen by antigen-presenting cells (APCs), which then process the transgene for major histocompatibility complex (MHC) presentation and immune clearance [52,53]. Furthermore, high-dose rAAV may elicit antigen-specific B-cell and T-cell responses. B cells can produce antibodies that target the rAAV capsid, while cytotoxic CD8^+^ T cells can recognize foreign peptides derived from the rAAV capsid, initiating the elimination of transduced cells. The formation of adaptive B-cell responses follows a mechanism similar to that of pre-existing humoral immunity [54,55]. Both of these immune responses can significantly impair therapeutic efficacy. High-dose rAAV has been implicated in treatment-emergent adverse events (TEAEs) associated with complement activation, including thrombocytopenia, thrombotic microangiopathy (TMA), liver enzyme elevation, and acute liver injury [56,57].

Current OA therapies, such as glucocorticoids, NSAIDs, and biologics, primarily focus on symptom management and disease slowing but are burdened by drawbacks like the necessity for repeated administrations, posing economic challenges to patients [4]. X-ray imaging technology is the sole clinically utilized and regulatory-accepted “gold standard” for diagnosing OA. However, when OA is diagnosed through imaging, the disease has already advanced significantly and is irreversible, demonstrating no responsiveness to any existing or experimental treatment modalities. Early disease identification through biomarkers can potentially improve the clinical outcomes associated with current treatments. Commonly employed OA biomarkers encompass cartilage matrix degradation products (e.g., CTX-II), inflammatory markers (e.g., IL-6 and TNF-α), metabolic markers (e.g., insulin and leptin), and bone remodeling biomarkers (e.g., NTX and BSAP). Regrettably, as van Spil has pointed out, “None of the current biochemical markers possess sufficient discriminatory power to aid in the diagnosis and prognosis of OA in individuals or small patient populations, nor do they exhibit consistent performance to serve as outcome measures in clinical trials [58,59]. 

OA is a multifaceted inflammatory and metabolic syndrome. Given its intricate pathogenesis, gene therapy strategies for osteoarthritis encompass diverse approaches, including the promotion of chondrocyte proliferation and growth, the inhibition of chondrocyte senescence and apoptosis, the enhancement of anabolic processes to increase extracellular matrix (ECM) production; and the prevention of inflammation, catabolism, and the synthesis of matrix-degrading enzymes [60]. Combination therapy leveraging multiple mechanisms can augment the therapeutic efficacy of AAV-IL-1Ra. Zhang et al. [61] demonstrated that the combined use of IL-1Ra and IL-10 exhibits a more potent inhibitory effect on cartilage destruction. Similarly, Adrianne Stone et al. [62] showed that inhibiting inflammation with IL-1Ra while concurrently using lubricin (PRG4) to promote cartilage protection results in superior preservation of articular cartilage compared to monotherapy. Gene therapy, in contrast, presents a promising alternative by enabling the direct delivery of therapeutic genes to the site of injury, stimulating the endogenous production of anti-inflammatory factors. The limited regenerative capacity of mammalian joints is advantageous in this context, as it supports long-term transgene expression without being diluted by cell proliferation, thus ensuring sustained therapeutic effects and minimizing tissue harm [63]. Localized administration further mitigates off-target effects and minimizes systemic side effects. In conclusion, the direct delivery of therapeutic genes to affected joints holds great potential as an effective long-term treatment strategy for OA patients, striving for substantial improvement or even curative outcomes.

## 5. Conclusions

Our study underscores the promising potential of AAV-mediated IL-1Ra gene therapy for treating osteoarthritis (OA), demonstrating statistically significant alleviation of joint swelling and a reduction in swelling diameter while concurrently preserving trabecular bone-related biomarkers in preclinical animal models. Importantly, we acknowledge that the immune response provoked by the AAV capsid itself can potentially modulate its therapeutic efficacy in OA. Consequently, when selecting high-titer AAV vectors for treating immune-mediated diseases, it is imperative to exercise caution and thoroughly evaluate the potential for the capsid to introduce more pronounced adverse effects.

## Data Availability

The original contributions presented in the study are included in the article/Supplementary Material, further inquiries can be directed to the corresponding author.

## Supplementary Materials

The following supporting information can be downloaded at: https://www.mdpi.com/article/10.3390/pharmaceutics16121518/s1, Figure S1: Representative photomicrographs of the right knee joint obtained from each group at the endpoint and stained with the Safranin O/Fast green staining. Figure S2: Knee joints were collected at the endpoint and examined using hematoxylin-eosin staining to assess inflammatory infiltration. Table S1: joint swelling. Table S2: Individual data on joint diameter and joint swelling.

## References

[1] GBD 2019 Diseases and Injuries Collaborators (2020). Global burden of 369 diseases and injuries in 204 countries and territories, 1990–2019: A systematic analysis for the Global Burden of Disease Study 2019. Lancet.

[2] Im G.-I., Kim T.-K. (2020). Regenerative Therapy for Osteoarthritis: A Perspective. Int. J. Stem Cells.

[3] Grässel S., Muschter D. (2020). Recent advances in the treatment of osteoarthritis. F1000Research.

[4] Cho Y., Jeong S., Kim H., Kang D., Lee J., Kang S.-B., Kim J.-H. (2021). Disease-modifying therapeutic strategies in osteoarthritis: Current status and future directions. Exp. Mol. Med..

[5] Martel-Pelletier J., Barr A.J., Cicuttini F.M., Conaghan P.G., Cooper C., Goldring M.B., Goldring S.R., Jones G., Teichtahl A.J., Pelletier J.-P. (2016). Osteoarthritis. Nat. Rev. Dis. Prim..

[6] Urban H., Little C.B. (2018). The role of fat and inflammation in the pathogenesis and management of osteoarthritis. Rheumatology.

[7] Arend W.P., Malyak M., Guthridge C.J., Gabay C. (1998). Interleukin-1 receptor antagonist: Role in biology. Annu. Rev. Immunol..

[8] Chevalier X., Goupille P., Beaulieu A.D., Burch F.X., Bensen W.G., Conrozier T., Loeuille D., Kivitz A.J., Silver D., Appleton B.E. (2009). Intraarticular injection of anakinra in osteoarthritis of the knee: A multicenter, randomized, double-blind, placebo-controlled study. Arthritis Care Res..

[9] Evans C.H., Kraus V.B., Setton L.A. (2013). Progress in intra-articular therapy. Nat. Rev. Rheumatol..

[10] Wang D., Tai P.W.L., Gao G. (2019). Adeno-associated virus vector as a platform for gene therapy delivery. Nat. Rev. Drug Discov..

[11] Li C., Samulski R.J. (2020). Engineering adeno-associated virus vectors for gene therapy. Nat. Rev. Genet..

[12] Ke X., Jiang H., Li Q., Luo S., Qin Y., Li J., Xie Q., Zheng Q. (2023). Preclinical evaluation of KH631, a novel rAAV8 gene therapy product for neovascular age-related macular degeneration. Mol. Ther..

[13] Levings R.S.W., Smith A.D., Broome T.A., Rice B.L., Gibbs E.P., Myara D.A., Hyddmark E.V., Nasri E., Zarezadeh A., Levings P.P. (2018). Self-Complementary Adeno-Associated Virus-Mediated Interleukin-1 Receptor Antagonist Gene Delivery for the Treatment of Osteoarthritis: Test of Efficacy in an Equine Model. Hum. Gene Ther. Clin. Dev..

[14] Shen W., Liu S., Ou L. (2022). rAAV immunogenicity, toxicity, and durability in 255 clinical trials: A meta-analysis. Front. Immunol..

[15] Qiao C., Yuan Z., Li J., He B., Zheng H., Mayer C., Xiao X. (2011). Liver-specific microRNA-122 target sequences incorporated in AAV vectors efficiently inhibits transgene expression in the liver. Gene Ther..

[16] Yang L., Liu Z., Chen G., Chen Z., Guo C., Ji X., Cui Q., Sun Y., Hu X., Zheng Y. (2024). MicroRNA-122-Mediated Liver Detargeting Enhances the Tissue Specificity of Cardiac Genome Editing. Circulation.

[17] Nguyen T.N., Sha S., Hong M.S., Maloney A.J., Barone P.W., Neufeld C., Wolfrum J., Springs S.L., Sinskey A.J., Braatz R.D. (2021). Mechanistic model for production of recombinant adeno-associated virus via triple transfection of HEK293 cells. Mol. Ther.-Methods Clin. Dev..

[18] Brand D.D., Latham K.A., Rosloniec E.F. (2007). Collagen-induced arthritis. Nat. Protoc..

[19] Cuzzocrea S., Mazzon E., Dugo L., Serraino I., Britti D., De Maio M., Caputi A.P. (2001). Absence of endogeneous interleukin-10 enhances the evolution of murine type-II collagen-induced arthritis. Eur. Cytokine Netw..

[20] Ke X., Xie Q., Luo S., Li Q., Zheng Q., Zhang Z. (2024). Intra-Articular Delivery of an AAV-Anti-TNF-α Vector Alleviates the Progress of Arthritis in a RA Mouse Model. Hum. Gene Ther..

[21] Luo S., Jiang H., Li Q., Qin Y., Yang S., Li J., Xu L., Gou Y., Zhang Y., Liu F. (2024). An adeno-associated virus variant enabling efficient ocular-directed gene delivery across species. Nat. Commun..

[22] Shaul O. (2017). How introns enhance gene expression. Int. J. Biochem. Cell Biol..

[23] Lacy-Hulbert A., Thomas R., Li X.-P., E Lilley C., Coffin R.S., Roes J. (2001). Interruption of coding sequences by heterologous introns can enhance the functional expression of recombinant genes. Gene Ther..

[24] Baier T., Jacobebbinghaus N., Einhaus A., Lauersen K.J., Kruse O. (2020). Introns mediate post-transcriptional enhancement of nuclear gene expression in the green microalga Chlamydomonas reinhardtii. PLoS Genet..

[25] Rose A.B., Carter A., Korf I., Kojima N. (2016). Intron sequences that stimulate gene expression in Arabidopsis. Plant Mol. Biol..

[26] Broderick L., Hoffman H.M. (2022). IL-1 and autoinflammatory disease: Biology, pathogenesis and therapeutic targeting. Nat. Rev. Rheumatol..

[27] Filipowicz W., Grosshans H. (2011). The liver-specific microRNA miR-122: Biology and therapeutic potential. Prog. Drug Res..

[28] Girard M., Jacquemin E., Munnich A., Lyonnet S., Henrion-Caude A. (2008). miR-122, a paradigm for the role of microRNAs in the liver. J. Hepatol..

[29] Jopling C. (2012). Liver-specific microRNA-122: Biogenesis and function. RNA Biol..

[30] Valdmanis P.N., Kim H.K., Chu K., Zhang F., Xu J., Munding E.M., Shen J., Kay M.A. (2018). miR-122 removal in the liver activates imprinted microRNAs and enables more effective microRNA-mediated gene repression. Nat. Commun..

[31] Lu H., Qu G., Yang X., Xu R., Xiao W. (2011). Systemic elimination of de novo capsid protein synthesis from replication-competent AAV contamination in the liver. Hum. Gene Ther..

[32] Bandiera S., Pfeffer S., Baumert T.F., Zeisel M.B. (2015). miR-122—A key factor and therapeutic target in liver disease. J. Hepatol..

[33] Lee J., Mou H., Ibraheim R., Liang S.-Q., Liu P., Xue W., Sontheimer E.J. (2019). Tissue-restricted genome editing in vivo specified by microRNA-repressible anti-CRISPR proteins. RNA.

[34] Yang Y.-S., Lin C., Ma H., Xie J., Kaplan F.S., Gao G., Shim J.-H. (2023). AAV-Mediated Targeting of the Activin A-ACVR1(R206H) Signaling in Fibrodysplasia Ossificans Progressiva. Biomolecules.

[35] Yin L., Keeler G.D., Zhang Y., Hoffman B.E., Ling C., Qing K., Srivastava A. (2021). AAV3-miRNA vectors for growth suppression of human hepatocellular carcinoma cells in vitro and human liver tumors in a murine xenograft model in vivo. Gene Ther..

[36] Xie J., Burt D.R., Gao G. (2015). Adeno-associated virus-mediated microRNA delivery and therapeutics. Semin. Liver Dis..

[37] Dismuke D.J., Tenenbaum L., Samulski R.J. (2014). Biosafety of recombinant adeno-associated virus vectors. Curr. Gene Ther..

[38] Ling Q., Herstine J.A., Bradbury A., Gray S.J. (2023). AAV-based in vivo gene therapy for neurological disorders. Nat. Rev. Drug Discov..

[39] Campochiaro P.A., Avery R., Brown D.M., Heier J.S., Ho A.C., Huddleston S.M., Jaffe G.J., Khanani A.M., Pakola S., Pieramici D.J. (2024). Gene therapy for neovascular age-related macular degeneration by subretinal delivery of RGX-314: A phase 1/2a dose-escalation study. Lancet.

[40] Wang J.-H., Gessler D.J., Zhan W., Gallagher T.L., Gao G. (2024). Adeno-associated virus as a delivery vector for gene therapy of human diseases. Signal Transduct. Target. Ther..

[41] Gerwin N., Bendele A., Glasson S., Carlson C. (2010). The OARSI histopathology initiative—Recommendations for histological assessments of osteoarthritis in the rat. Osteoarthr. Cartil..

[42] Thysen S., Luyten F.P., Lories R.J.U. (2015). Targets, models and challenges in osteoarthritis research. Dis. Model. Mech..

[43] Wu F., Luo S., Zhang Y., Ou Y., Wang H., Guo Z., He C., Bai S., He P., Jiang M. (2022). Single-shot AAV-vectored vaccine against SARS-CoV-2 with fast and long-lasting immunity. Acta Pharm. Sin. B.

[44] Wu I., Zeng A., Greer-Short A., Aycinena J.A., Tefera A.E., Shenwai R., Farshidfar F., Van Pell M., Xu E., Reid C. (2024). AAV9:PKP2 improves heart function and survival in a Pkp2-deficient mouse model of arrhythmogenic right ventricular cardiomyopathy. Commun. Med..

[45] Schulz M., Levy D., Petropoulos C.J., Bashirians G., Winburn I., Mahn M., Somanathan S., Cheng S.H., Byrne B.J. (2023). Binding and neutralizing anti-AAV antibodies: Detection and implications for rAAV-mediated gene therapy. Mol. Ther..

[46] Wang G., Evans C.H., Benson J.M., Hutt J.A., Seagrave J., Wilder J.A., Grieger J.C., Samulski R.J., Terse P.S. (2016). Safety and biodistribution assessment of sc-rAAV2.5IL-1Ra administered via intra-articular injection in a mono-iodoacetate-induced osteoarthritis rat model. Mol. Ther.-Methods Clin. Dev..

[47] Levings R.S.W., Broome T.A., Smith A.D., Rice B.L., Gibbs E.P., Myara D.A., Hyddmark E.V., Nasri E., Zarezadeh A., Levings P.P. (2018). Gene Therapy for Osteoarthritis: Pharmacokinetics of Intra-Articular Self-Complementary Adeno-Associated Virus Interleukin-1 Receptor Antagonist Delivery in an Equine Model. Hum. Gene Ther. Clin. Dev..

[48] Xie J., Zhang D., Lin Y., Yuan Q., Zhou X. (2018). Anterior Cruciate Ligament Transection-Induced Cellular and Extracellular Events in Menisci: Implications for Osteoarthritis. Am. J. Sports Med..

[49] Martino A.T., Suzuki M., Markusic D.M., Zolotukhin I., Ryals R.C., Moghimi B., Ertl H.C.J., Muruve D.A., Lee B., Herzog R.W. (2011). The genome of self-complementary adeno-associated viral vectors increases Toll-like receptor 9-dependent innate immune responses in the liver. Blood.

[50] Ashley S.N., Somanathan S., Giles A.R., Wilson J.M. (2019). TLR9 signaling mediates adaptive immunity following systemic AAV gene therapy. Cell Immunol..

[51] Zhu J., Huang X., Yang Y. (2009). The TLR9-MyD88 pathway is critical for adaptive immune responses to adeno-associated virus gene therapy vectors in mice. J. Clin. Investig..

[52] Muhuri M., Zhan W., Maeda Y., Li J., Lotun A., Chen J., Sylvia K., Dasgupta I., Arjomandnejad M., Nixon T. (2021). Novel Combinatorial MicroRNA-Binding Sites in AAV Vectors Synergistically Diminish Antigen Presentation and Transgene Immunity for Efficient and Stable Transduction. Front. Immunol..

[53] Xiao Y., Muhuri M., Li S., Qin W., Xu G., Luo L., Li J., Letizia A.J., Wang S.K., Chan Y.K. (2019). Circumventing cellular immunity by miR142-mediated regulation sufficiently supports rAAV-delivered OVA expression without activating humoral immunity. J. Clin. Investig..

[54] Ertl H.C.J. (2021). T Cell-Mediated Immune Responses to AAV and AAV Vectors. Front. Immunol..

[55] Ertl H.C.J. (2022). Immunogenicity and toxicity of AAV gene therapy. Front. Immunol..

[56] Chand D.H., Zaidman C., Arya K., Millner R., Farrar M.A., Mackie F.E., Goedeker N.L., Dharnidharka V.R., Dandamudi R., Reyna S.P. (2021). Thrombotic Microangiopathy Following Onasemnogene Abeparvovec for Spinal Muscular Atrophy: A Case Series. J. Pediatr..

[57] Mullard A. (2021). Gene therapy community grapples with toxicity issues, as pipeline matures. Nat. Rev. Drug Discov..

[58] Hunter D.J., Guermazi A., Roemer F., Zhang Y., Neogi T. (2013). Structural correlates of pain in joints with osteoarthritis. Osteoarthr. Cartil..

[59] Lafeber F.P., van Spil W.E. (2013). Osteoarthritis year 2013 in review: Biomarkers; reflecting before moving forward, one step at a time. Osteoarthr. Cartil..

[60] Li X., Shen L., Deng Z., Huang Z. (2023). New treatment for osteoarthritis: Gene therapy. Precis. Clin. Med..

[61] Zhang X., Mao Z., Yu C. (2004). Suppression of early experimental osteoarthritis by gene transfer of interleukin-1 receptor antagonist and interleukin-10. J. Orthop. Res..

[62] Stone A., Grol M.W., Ruan M.Z.C., Dawson B., Chen Y., Jiang M.-M., Song I.-W., Jayaram P., Cela R., Gannon F. (2019). Combinatorial Prg4 and Il-1ra Gene Therapy Protects Against Hyperalgesia and Cartilage Degeneration in Post-Traumatic Osteoarthritis. Hum. Gene Ther..

[63] Karuppal R. (2017). Current concepts in the articular cartilage repair and regeneration. J. Orthop..

