# Supplementary material for: The Intra-Articular Delivery of a Low-Dose Adeno-Associated Virus-IL-1 Receptor Antagonist Vector Alleviates the Progress of Arthritis in an Osteoarthritis Rat Model
Source: Pharmaceutics. 2024 Nov 25;16(12):1518. doi: 10.3390/pharmaceutics16121518 (PMC11728506; doi:10.3390/pharmaceutics16121518)
Supplement: Supplementary file 1 [file pharmaceutics-16-01518-s001.zip › pharmaceutics-3243910-supplementary.pdf]

Supplementary Material

Supplementary Material S1

No.0 Sequence

atggaaatctgcagaggcctccgctcacacctaatcactctcctcctcttcctgttccattcagagacgatctgcagaccctctggc
agaaagtccagcaagatgcaggccttcagaatctgggatgtgaaccagaaaacattctacctgaggaacaatcagctggtgg
caggctacctgcagggccccaatgtgaacctggaggagaagattgatgtggtgcccattgagccccatgctctgtttctgggca
tccatggtggcaagatgtgcctgagctgtgtgaagtctggggatgagacaagactgcagctggaagctgtgaacatcacaga
cctgtctgagaacagaaagcaggacaagagatttgccttcattagatctgactctggccccaccaccagctttgagtctgctgctt
gtcctggctggttcctgtgcactgccatggaggcagaccagcctgtgtctctgaccaacatgcctgatgagggtgtcatggtcac
caagttctacttccaagaggatgagtga

No.1 Sequence

atggaaatctgcagaggcctccgctcacacctaatcactctcctcctcttcctgttccattcagagacgatctgcagaccctctggc
agaaagtccagcaagatgcaggccttcagaatctgggatgtgaaccagaaaacattctacctgaggtgagtatctcagggatc
cagacatggggatatgggaggtgcctctgatcccagggctcactgtgggtctctctgttcacaggaacaatcagctggtggca
ggctacctgcagggccccaatgtgaacctggaggagaagattgatgtggtgcccattgagccccatgctctgtttctgggcatc
catggtggcaagatgtgcctgagctgtgtgaagtctggggatgagacaagactgcagctggaagctgtgaacatcacagacc
tgtctgagaacagaaagcaggtaagtatcaaggttacaagacaggtttaaggagaccaatagaaactgggcttgtcgagaca
gagaagactcttgcgtttctgataggcacctattggtcttactgacatccactttgcctttctctccacaggacaagagatttgccttc
attagatctgactctggccccaccaccagctttgagtctgctgcttgtcctggctggttcctgtgcactgccatggaggcagacca
gcctgtgtctctgaccaacatgcctgatgagggtgtcatggtcaccaagttctacttccaagaggatgagtga

No.2 Sequence

atggaaatctgcagaggcctccgctcacacctaatcactctcctcctcttcctgttccattcagagacgatctgcagaccctctggc
agaaagtccagcaagatgcaggccttcagaatctgggatgtgaaccagaaaacattctacctgaggtgagtatctcagggatc
cagacatggggatatgggaggtgcctctgatcccagggctcactgtgggtctctctgttcacaggaacaatcagctggtggca
ggctacctgcagggccccaatgtgaacctggaggagaagattgatgtggtgcccattgagccccatgctctgtttctgggcatc
catggtggcaagatgtgcctgagctgtgtgaagtctggggatgagacaagactgcagctggaagctgtgaacatcacagacc

tgtctgagaacagaaagcaggtaagtatcaaggttacaagacaggtttaaggagaccaatagaaactgggcttgtcgagaca
gagaagactcttgcgtttctgataggcacctattggtcttactgacatccactttgcctttctctccacaggacaagagatttgccttc
attagatctgactctggccccaccaccagctttgagtctgctgcttgtcctggctggttcctgtgcactgccatggaggcagacca
gcctgtgtctctgaccaacatgcctgatgagggtgtcatggtcaccaagttctacttccaagaggatgagtga

No.3 Sequence

atggaaatctgcagaggcctgagaagccacctgatcaccctgctgctgttcctgttccacagcgagacaatctgcaggtgagta
tctcagggatccagacatggggatatgggaggtgcctctgatcccagggctcactgtgggtctctctgttcacaggcccagcgg
cagaaagtccagcaagatgcaggccttccggatctgggacgtgaaccagaaaaccttctacctgcggaacaatcagctggtg
gccggctatctgcagggccccaatgtgaacctggaggagaagatcgacgtggtgcccatcgagccccacgctctgtttctggg
aattcacggcggcaagatgtgcctgagctgtgtgaagtctggcgacgagacacggctgcagctggaagccgtgaacatcacc
gacctgagcgagaaccggaagcaggtaagtttagtctttttgtcttttatttcaggtcccggatccggtggtggtgcaaatcaaag
aactgctcctcagtggatgttgcctttacttctaggacaagagattcgccttcatcagaagcgacagcggccccaccaccagcttt
gagtctgctgcttgccctggctggttcctgtgtacagccatggaggccgaccagcctgtgtctctgaccaacatgcctgacgagg
gcgtgatggtcaccaagttctacttccaagaggacgagtga

No.4 Sequence

atggaaatctgcagaggcctgagaagccacctgatcaccctgctgctgttcctgttccacagcgagacaatctgcaggtgagta
tctcagggatccagacatggggatatgggaggtgcctctgatcccagggctcactgtgggtctctctgttcacaggcccagcgg
cagaaagtccagcaagatgcaggccttccggatctgggacgtgaaccagaaaaccttctacctgcggaacaatcagctggtg
gccggctatctgcagggccccaatgtgaacctggaggagaagatcgacgtggtgcccatcgagccccacgctctgtttctggg
aattcacggcggcaagatgtgcctgagctgtgtgaagtctggcgacgagacacggctgcagctggaagccgtgaacatcacc
gacctgagcgagaaccggaagcaggtaagtttagtctttttgtcttttatttcaggtcccggatccggtggtggtgcaaatcaaag
aactgctcctcagtggatgttgcctttacttctaggacaagagattcgccttcatcagaagcgacagcggccccaccaccagcttt
gagtctgctgcttgccctggctggttcctgtgtacagccatggaggccgaccagcctgtgtctctgaccaacatgcctgacgagg
gcgtgatggtcaccaagttctacttccaagaggacgagtga

No.5 Sequence

atggaaatctgcagaggcctgagaagccacctgatcaccctgctgctgttcctgttccacagcgagacaatctgcaggtgagta
tctcagggatccagacatggggatatgggaggtgcctctgatcccagggctcactgtgggtctctctgttcacaggcccagcgg

cagaaagtccagcaagatgcaggccttccggatctgggacgtgaaccagaaaaccttctacctgcggaacaatcagctggtg
gccggctatctgcagggccccaatgtgaacctggaggagaagatcgacgtggtgcccatcgagccccacgctctgtttctggg
aattcacggcggcaagatgtgcctgagctgtgtgaagtctggcgacgagacacggctgcagctggaagccgtgaacatcacc
gacctgagcgagaaccggaagcaggtaagtatcaaggttacaagacaggtttaaggagaccaatagaaactgggcttgtcg
agacagagaagactcttgcgtttctgataggcacctattggtcttactgacatccactttgcctttctctccacaggacaagagattc
gccttcatcagaagcgacagcggccccaccaccagctttgagtctgctgcttgccctggctggttcctgtgtacagccatggagg
ccgaccagcctgtgtctctgaccaacatgcctgacgagggcgtgatggtcaccaagttctacttccaagaggacgagtga

No.6 Sequence

Atggaaatctgcagaggcctccgctcacacctaatcactctcctcctcttcctgttccattcagagacgatctgcagaccctctggc
agaaagtccagcaagatgcaggccttcagaatctgggatgtgaaccagaaaacattctacctgaggtgagtatctcagggatc
cagacatggggatatgggaggtgcctctgatcccagggctcactgtgggtctctctgttcacaggaacaatcagctggtggca
ggctacctgcagggccccaatgtgaacctggaggagaagattgatgtggtgcccattgagccccatgctctgtttctgggcatc
catggtggcaagatgtgcctgagctgtgtgaagtctggggatgagacaagactgcagctggaagctgtgaacatcacagacc
tgtctgagaacagaaagcaggacaagagatttgccttcattagatctgactctggccccaccaccagctttgagtctgctgcttgt
cctggctggttcctgtgcactgccatggaggcagaccagcctgtgtctctgaccaacatgcctgatgagggtgtcatggtcacca
agttctacttccaagaggatgagtga

Supplementary Table S1 joint swelling (%, Mean ± SEM)

Group	N	Day 0	Day 14	Day 28	Day 42	Day 56
Sham surgery	8	2.03±0.98	0.22±0.70	0.91±1.02	0.07±0.48	1.89±1.14
Vehicle Control	10	22.50±2.71	17.32±2.31	28.77±2.84	28.65±3.22	31.74±4.85
High-dose AAV5	10	22.49±2.68	16.09±1.90	21.27±2.21	21.91±3.05	30.77±2.76
Low-dose AAV5	10	22.48±2.68	15.42±1.88	21.82±2.14	20.03±3.29	20.62±3.03
High-dose AAV9	10	22.50±2.70	15.53±1.50	26.89±1.67	25.51±2.51	24.68±2.22
Low-dose AAV9	10	22.42±2.74	17.29±2.02	13.80±2.66	13.29±3.11	13.88±2.71

Supplementary Table S2 Individual data on joint diameter and joint swelling

Group	No.	Day 0			Day 14			Day 28			Day 42			Day 56		
		LD (mm)	RD (mm)	Swelling %	LD (mm)	RD (mm)	Swelling %	LD (mm)	RD (mm)	Swelling %	LD (mm)	RD (mm)	Swelling %	LD (mm)	RD (mm)	Swelling %
Sham surgery	1F0101	9.01	9.05	0.44	10.85	10.89	0.37	10.83	10.43	-3.69	12.37	12.41	0.32	12.42	12.58	1.29
	1F0102	9.21	9.11	-1.09	10.87	10.77	-0.92	11.01	11.47	4.18	12.28	12.34	0.49	11.42	12.06	5.60
	1F0103	9.27	9.27	0.00	10.44	10.12	-3.07	11.46	11.23	-2.01	12.19	12.14	-0.41	11.83	12.50	5.66
	1F0104	9.59	9.71	1.25	10.50	10.60	0.95	12.13	12.17	0.33	11.40	11.37	-0.26	10.49	10.09	-3.81
	1F0201	9.58	9.87	3.03	10.17	10.27	0.98	11.36	11.91	4.84	11.24	11.52	2.49	11.37	11.82	3.96
	1F0202	8.00	8.61	7.62	9.49	9.43	-0.63	12.23	12.51	2.29	12.43	12.13	-2.41	11.33	11.27	-0.53
	1F0203	9.33	9.43	1.07	10.16	10.18	0.20	12.04	12.07	0.25	12.14	12.14	0.00	11.56	11.84	2.42
	1F0204	9.56	9.93	3.87	11.02	11.33	3.87	13.19	13.33	1.06	13.09	13.14	0.38	13.86	13.93	0.51
Vehicle Control	2F0101	8.54	11.88	39.11	12.04	13.52	12.29	10.77	13.94	29.43	10.11	14.70	45.40	10.47	14.33	36.87
	2F0102	9.08	11.89	30.95	12.23	13.42	9.73	10.19	14.21	39.45	10.17	14.16	39.23	10.05	14.65	45.77
	2F0103	9.63	11.82	22.74	10.79	12.53	16.13	10.20	13.18	29.22	10.85	13.44	23.87	10.77	14.74	36.86
	2F0104	9.72	11.19	15.12	11.31	13.57	19.98	10.43	14.46	38.64	11.81	13.64	15.50	11.42	14.96	31.00
	2F0105	9.00	10.89	21.00	10.28	13.77	33.95	12.55	14.80	17.93	12.14	14.92	22.90	11.43	14.94	30.71
	2F0201	9.90	11.05	11.62	11.12	13.01	17.00	9.81	14.02	42.92	10.22	14.09	37.87	11.48	14.33	24.83
	2F0202	9.53	11.11	16.58	12.00	13.23	10.25	11.98	14.57	21.62	11.22	14.46	28.88	10.21	14.97	46.62
	2F0203	8.79	11.30	28.56	11.78	13.46	14.26	12.40	14.69	18.47	12.48	14.95	19.79	10.31	14.43	39.96
	2F0204	8.72	10.88	24.77	10.59	12.22	15.39	11.50	14.66	27.48	11.04	14.90	34.96	11.11	14.68	32.13
	2F0205	9.06	10.38	14.57	10.43	12.96	24.26	12.13	14.86	22.51	12.14	14.34	18.12	11.90	11.02	-7.39
High-dose AAV5	3F0101	9.62	10.72	11.43	11.04	12.91	16.94	11.11	14.15	27.36	11.35	14.65	29.06	11.91	14.15	18.81
	3F0102	9.05	11.75	29.83	10.37	12.64	21.89	11.03	14.53	31.73	11.54	14.65	26.95	11.15	14.70	31.84
	3F0103	8.89	10.18	14.51	10.62	13.56	27.68	13.00	15.58	19.85	13.40	15.70	17.16	10.05	14.94	48.66
	3F0104	9.55	11.02	15.39	11.17	12.56	12.44	11.06	12.90	16.64	11.86	12.24	3.20	11.95	14.92	24.85

	3F0105	8.53	11.21	31.42	12.00	13.00	8.33	11.88	14.28	20.20	11.12	14.73	32.46	10.33	14.50	40.37
	3F0201	8.05	11.06	37.39	10.70	11.87	10.93	10.80	14.40	33.33	10.48	14.34	36.83	11.41	14.62	28.13
	3F0202	8.04	10.16	26.37	11.19	13.44	20.11	10.95	12.67	15.71	10.68	12.64	18.35	10.98	14.66	33.52
	3F0203	9.64	11.64	20.75	10.78	12.19	13.08	11.72	13.37	14.08	11.75	13.75	17.02	11.04	14.51	31.43
	3F0204	9.55	11.58	21.26	10.86	12.07	11.14	10.44	12.31	17.91	10.32	12.35	19.67	11.61	14.20	22.31
	3F0205	8.96	10.44	16.52	10.08	11.93	18.35	10.39	12.04	15.88	10.46	12.38	18.36	11.31	14.45	27.76
Low-dose AAV5	4F0101	9.61	11.70	21.75	10.68	13.16	23.22	10.26	14.20	38.40	11.02	14.73	33.67	10.68	10.41	-2.53
	4F0102	9.00	10.27	14.11	11.54	13.32	15.42	12.29	14.76	20.10	12.88	14.66	13.82	11.63	14.10	21.24
	4F0103	8.20	11.17	36.22	11.28	12.51	10.90	12.19	14.72	20.75	11.42	14.46	26.62	11.54	14.20	23.05
	4F0104	8.68	10.14	16.82	10.66	12.20	14.45	12.18	14.49	18.97	12.54	14.10	12.44	11.80	14.51	22.97
	4F0105	9.07	10.74	18.41	11.76	12.63	7.40	10.71	13.64	27.36	12.39	13.48	8.80	11.27	14.49	28.57
	4F0201	9.27	10.34	11.54	11.30	12.65	11.95	10.68	12.75	19.38	11.84	12.77	7.85	11.31	14.58	28.91
	4F0202	8.88	11.55	30.07	9.91	12.29	24.02	11.20	13.08	16.79	11.18	13.68	22.36	10.78	13.26	23.01
	4F0203	8.14	10.33	26.90	11.14	13.65	22.53	11.29	13.00	15.15	11.08	12.64	14.08	11.74	13.19	12.35
	4F0204	8.98	10.44	16.26	11.72	13.44	14.68	11.91	14.05	17.97	11.98	14.68	22.54	10.20	13.16	29.02
	4F0205	9.03	11.98	32.67	11.42	12.52	9.63	11.58	14.28	23.32	10.81	14.93	38.11	11.85	14.17	19.58
High-dose AAV9	5F0101	8.01	10.28	28.34	10.16	11.96	17.72	10.52	12.74	21.10	10.91	13.48	23.56	10.40	13.29	27.79
	5F0102	9.05	10.14	12.04	10.56	11.74	11.17	10.55	13.44	27.39	10.27	13.63	32.72	10.86	14.00	28.91
	5F0103	8.03	10.02	24.78	10.96	11.77	7.39	10.64	13.12	23.31	10.33	13.50	30.69	10.66	13.10	22.89
	5F0104	8.91	10.51	17.96	10.52	11.72	11.41	10.72	14.81	38.15	10.99	12.19	10.92	10.02	13.19	31.64
	5F0105	8.91	10.21	14.59	10.01	11.42	14.09	11.19	13.79	23.24	10.59	13.79	30.22	10.51	13.72	30.54
	5F0201	8.98	10.90	21.38	10.66	12.19	14.35	10.06	12.23	21.57	10.22	11.62	13.70	10.47	12.76	21.87
	5F0202	8.62	11.50	33.41	11.33	13.69	20.83	11.22	14.59	30.04	10.24	13.64	33.20	11.10	13.28	19.64
	5F0203	8.99	10.37	15.35	11.19	13.02	16.35	10.90	14.34	31.56	10.80	13.85	28.24	10.35	13.90	34.30
	5F0204	9.86	11.74	19.07	11.15	13.62	22.15	11.61	14.49	24.81	10.89	13.22	21.40	11.54	13.17	14.12
	5F0205	8.46	11.68	38.06	10.39	12.45	19.83	10.69	13.65	27.69	10.15	13.24	30.44	11.39	13.11	15.10
Low-dose AAV9	6F0101	9.48	11.70	23.42	10.92	12.85	17.67	10.31	11.12	7.86	11.42	12.84	12.43	10.46	12.45	19.02
	6F0102	8.92	11.68	30.94	11.59	13.54	16.82	11.64	12.53	7.65	11.44	12.84	12.24	11.68	12.31	5.39

6F0103	9.85	11.88	20.61	10.60	12.33	16.32	10.43	12.64	21.19	10.07	12.09	20.06	10.98	12.41	13.02
6F0104	8.87	10.42	17.47	10.95	12.21	11.51	10.82	11.89	9.89	10.30	11.05	7.28	11.00	12.43	13.00
6F0105	9.03	10.35	14.62	10.15	12.66	24.73	11.24	13.05	16.10	11.89	12.97	9.08	11.49	12.36	7.57
6F0201	8.93	10.15	13.66	11.47	13.00	13.34	11.76	11.86	0.85	11.05	11.19	1.27	11.96	12.01	0.42
6F0202	8.32	11.61	39.54	10.46	13.22	26.39	11.32	13.59	20.05	11.86	12.67	6.83	10.89	12.94	18.82
6F0203	9.82	12.00	22.20	11.55	12.51	8.31	11.24	13.40	19.22	10.54	13.86	31.50	11.79	13.81	17.13
6F0204	8.06	10.41	29.16	10.43	13.12	25.79	10.41	11.14	7.01	11.83	12.45	5.24	11.19	12.65	13.05
6F0205	9.07	10.21	12.57	11.61	13.01	12.06	11.04	14.15	28.17	11.03	14.00	26.93	11.33	14.88	31.33

Note:

1. LD and RD represent the diameters of the left and right knee joints, respectively, unit:mm.
2. The right side is the surgically modeled side, and the left side is the control side.

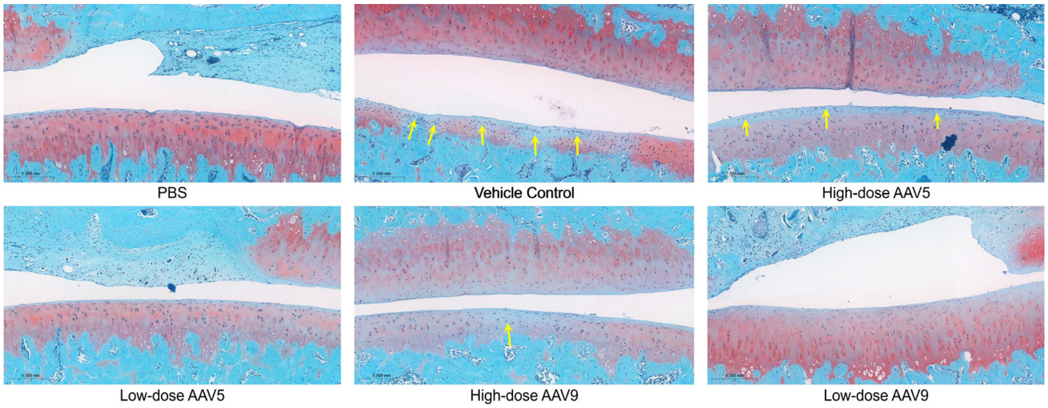

Supplementary Figure S1. Representative photomicrographs of the right knee joint obtained from each group at the endpoint and stained with the Safranin O/Fast green staining. Red color indicates articular cartilage. Green color indicates subchondral bone. In the vehicle control group, the articular cartilage exhibited surface irregularities, decreased faintly red chondrocytes (as indicated by yellow arrows), reduced extracellular matrix, irregular arrangement, and tissue debris. In the low-dose AAV9 group, the knee joint exhibited a comparable morphology to that observed in the negative control group (PBS), displaying a regular arrangement of richly stained red chondrocytes. However, in the high-dose AAV5 and AAV9 groups, a decrease in the number of faintly red chondrocytes was noted (as indicated by yellow arrows). In the low-dose AAV5 groups, no obvious chondrocyte reduction was observed.

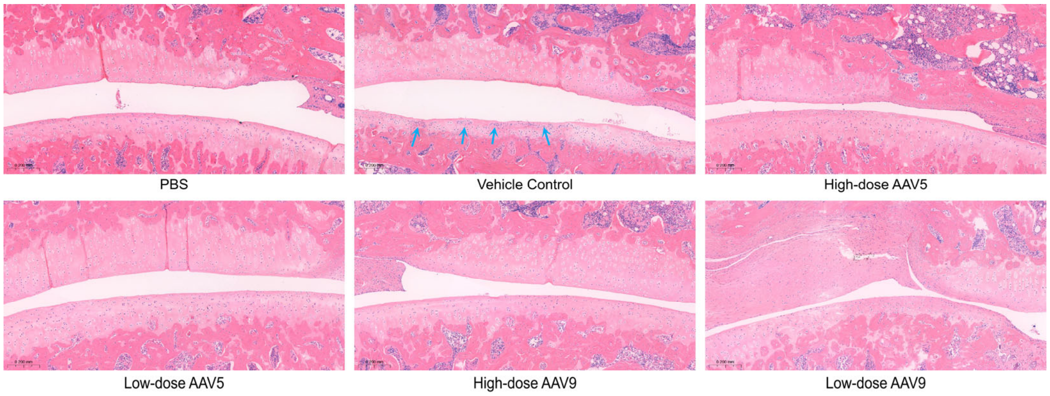

Supplementary Figure S2. Knee joints were collected at the endpoint and examined using hematoxylin-eosin staining to assess inflammatory infiltration. The vehicle control group exhibited evidence of tissue damage, structural disruption, and the

infiltration of neutrophils and lymphocytes (as indicated by the blue arrows). The other groups exhibited no notable inflammatory infiltration.
